# Therapeutic suppression of premature termination codons: Mechanisms and clinical considerations (Review)

**DOI:** 10.3892/ijmm.2014.1809

**Published:** 2014-06-17

**Authors:** JOHN KARIJOLICH, YI-TAO YU

**Affiliations:** 1 Department of Plant and Microbial Biology, University of California, Berkeley, CA 94720, USA; 2 Department of Biochemistry and Biophysics, University of Rochester School of Medicine and Dentistry, Rochester, NY 14642, USA

**Keywords:** stop codons, nonsense suppression (read-through), aminoglycosides, ptc124, pseudouridylation

## Abstract

An estimated one-third of genetic disorders are the result of mutations that generate premature termination codons (PTCs) within protein coding genes. These disorders are phenotypically diverse and consist of diseases that affect both young and old individuals. Various small molecules have been identified that are capable of modulating the efficiency of translation termination, including select antibiotics of the aminoglycoside family and multiple novel synthetic molecules, including PTC124. Several of these agents have proved their effectiveness at promoting nonsense suppression in preclinical animal models, as well as in clinical trials. In addition, it has recently been shown that box H/ACA RNA-guided peudouridylation, when directed to modify PTCs, can also promote nonsense suppression. In this review, we summarize our current understanding of eukaryotic translation termination and discuss various methods for promoting the read-through of disease-causing PTCs, as well as the current obstacles that stand in the way of using the discussed agents broadly in clinical practice.

## 1. Introduction

**Figure 1:**
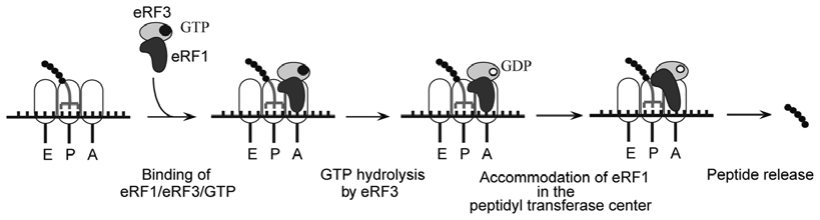
Translation termination in eukaryotes. Eukaryotic release factor 1 (eRF1) and eRF3 bind to ribosomal pre-termination complexes as an eRF1•eRF3•GTP ternary complex. GTP hydrolysis facilitates the positioning of the GGQ motif of eRF1 into the peptidyl transferase center. eRF1 induces peptide release.

In most genetic systems, including humans, translation termination occurs when one of three stop (nonsense) codons (UAA, UAG and UGA) enters the ribosomal A-site (1–3). In contrast to the recognition of sense codons which is carried out by transfer RNA (tRNA), the recognition of stop codons is mediated by extra-ribosomal proteins known as class 1 release factors (RFs). Subsequent to the recognition of the nonsense codon, release factors trigger the hydrolysis of the ester bond between the nascent polypeptide chain and the tRNA in the ribosomal P-site, resulting in translation termination (3) (Fig. 1).

Translation termination is not a perfect process and its efficiency depends on competition between the recognition of the stop codon by a release factor and decoding of the stop codon by a near-cognate tRNA (paired using two of the three bases). The fact that translation termination is not 100% efficient results in a low level of natural nonsense suppression or translational read-through of the termination codon. Translational read-through results in an amino acid being incorporated in place of the stop codon and the synthesis of a C-terminally extended protein that terminates at the next stop codon present in the same reading frame; all termination codons, whether natural or premature exhibit low levels of translational read-through. Multiple factors contribute to the efficiency of translational read-through, including the identity of the nucleotides 5′ and 3′ of the termination codon (4). Additionally, the termination codons themselves mediate translation termination with varying efficiencies (UAA ≥ UAG ≥ UGA), which is inversely correlated with the extent of natural read-through that occurs at each nonsense codon (UGA ≥ UAG ≥ UAA) (4,5).

While understanding the mechanism of protein synthesis has always attracted considerable interest, the realization that premature termination codons (PTCs) contribute to human pathology has significantly increased the attention given to the mechanism of translation termination and the mechanisms capable of promoting translational read-through (or nonsense suppression). PTCs have been implicated in several human diseases with ~2,400 distinct genetic disorders having at least one causative PTC allele (6). Additionally, a recent meta-analysis of the Human Gene Mutation Database (HGMD) estimated that ~11% of HGMD lesions responsible for inherited disorders are nonsense generating mutations (7). In addition to the C-terminally truncated protein that is synthesized as a result of the PTC, the presence of a PTC within a messenger RNA (mRNA) is often, though not always, accompanied with an increased rate of mRNA decay via nonsense mediated mRNA decay (NMD) (8). Thus, as a result of the reduction in gene expression, coupled with the synthesis of a C-terminally truncated protein product which may be harmful to cells, nonsense mutations that trigger NMD are more likely to lead to a disease phenotype (7).

Several small molecules have been identified that are capable of modulating the efficiency of translation termination (9, and references therein). More recently, novel strategies based on targeting the nucleotides of the PTC for post-transcriptional modification have been developed. While these methods are still in their infancy, they represent an exciting new avenue towards the development of therapeutics capable of specifically suppressing PTCs. In this review, we summarize our current understanding of the mechanism of translation termination and discuss multiple strategies currently being investigated to promote translational read-through of PTCs.

## 2. Mechanism of translation termination

Translation termination occurs when a stop codon enters the ribosomal A-site. For simplicity, termination can be thought of as two distinct steps, stop codon recognition and peptide release. In eukaryotes, translation termination is mediated by eukaryotic release factor 1 (eRF1), which is responsible for stop codon recognition and triggering peptide release, and eRF3, a GTPase that stimulates eRF1-mediated peptide release (10,11). eRF1, in turn, stabilizes binding of GTP to eRF3 so that they form a stable ternary complex (12,13) (Fig. 1).

X-ray crystallographic and nuclear magnetic resonance (NMR) data of the RFs have provided significant insight regarding their mechanism of action (14–16). Of note, eRF1, responsible for stop codon recognition, adopts a fold resembling that of a tRNA and is comprised of three distinct domains, namely N-terminal (N), middle (M) and C-terminal (C) (16). Although the mechanism by which eRF1 engages each of the three stop codons is unknown, mutational and genetic analyses have identified an essential role in this process for the GTS_31–33_, TASNIKS_58–64_ and YxCxxxF_125–131_ motifs located within the N-domain (16–26). Upon stop codon recognition the highly conserved Gly-Gly-Gln (GGQ) motif of eRF1, located within the M domain, is positioned within the peptidyl transferase center resulting in a rearrangement of rRNA, allowing for the entry of a water molecule and subsequent triggering of peptidyl-tRNA hydrolysis (27–30). The rearrangement and correct positioning of the GGQ motif is partially driven by GTP hydrolysis by eRF3. In line with this, in the presence of eRF3, eRF1 adopts a structure more ‘tRNA-like’ than that observed for free eRF1 (23). It is important to note that although the GGQ motif of eRF1 is inserted into the peptidyl transferase center and is critical for peptide hydrolysis, a water molecule actually serves as the nucleophile in the hydrolysis of the peptidyl-tRNA ester bond. The inclusion of a water molecule in the peptidyl transferase center during termination makes this reaction particularly distinct from polypeptide elongation where ribosomes employ mechanisms to keep water out during amide formation (31).

eRF3 consists of two domains, the N- and C-terminal. The C-terminal domain of eRF3 is responsible for its interaction with eRF1. Additionally, the C-terminal domain contains a GTP-binding domain and β-barrel domains 2 and 3, which are homologous to translation elongation factors EF-Tu and eEF1A (14). The N-terminal domain of eRF3 appears not to be required for translation termination (23,32,33). eRF3 can bind GTP independently of eRF1; however, stimulation of eRF3 GTPase activity requires both eRF1 and the ribosome. Of note, however, a stop codon is not required for GTPase stimulation. eRF1 and eRF3 bind to ribosomal pre-termination complexes as an eRF1•eRF3•GTP ternary complex with peptide release being dependent on eRF3-mediated GTP hydrolysis (34). GTP hydrolysis releases eRF1’s M domain from eRF3 enabling the correct positioning of the GGQ motif within the peptidyl transferase center, thereby promoting peptide hydrolysis (16).

## 3. PTC suppressive therapeutics

According to the National Organization for Rare Disorders (NORD), there are over 7,000 rare genetic diseases. Additionally, as mentioned above, ~2,400 distinct genetic disorders have at least one causative PTC allele. Thus, there is an immense need for pharmacological agents that are capable of promoting nonsense suppression. Ultimately, the goal of suppression therapy is to enhance the ability of near-cognate aminoacyl tRNAs to out compete release factors for the binding to PTCs, resulting in the incorporation of an amino acid at the PTC. Through increasing the frequency that PTCs are read-through, it is possible that enough functional full-length protein can be produced to reduce disease severity. Below, we review various strategies that have been shown to promote the suppression of PTCs.

### Aminoglycosides

**Figure 2:**
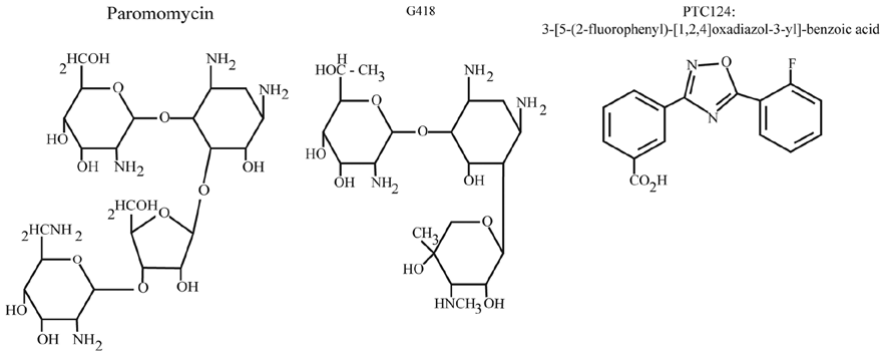
Structures of agents shown to possess nonsense suppressive properties.

In 1944 antibiotics of the aminoglycoside family were first isolated from soil bacteria (35,36). Structurally, there are two major classes of aminoglycosides, characterized by either 4,5- or 4,6-disubstituted 2-deoxystreptamine linked to an amino sugar backbone (37) (Fig. 2). The antibacterial activity of aminoglycosides is well established exhibiting broad activity against many Gram-negative bacteria, select Gram-positive bacteria, and non-tuberculous mycobacteria (38). The antibacterial activity of aminoglycosides is a result of their binding to the decoding site of the bacterial 16S rRNA (39). The decoding center possesses a proofreading function where it monitors base pairing between the mRNA codon and incoming aminoacyl tRNAs (40,41). Through interacting with the decoding center, aminoglycosides reduce the fidelity of the proofreading process resulting in increased misincorporation of near-cognate aminoacyl tRNAs into the ribosomal A-site, leading to an accumulation of non-functional or truncated bacterial proteins and culminating in bacterial cell death.

Although the decoding center of the ribosome is fairly well conserved between prokaryotic and eukaryotic organisms, the nucleotides responsible for the high affinity binding of aminoglycosides to the prokaryotic 16S rRNA (A1408 and G1491) are absent in the mammalian decoding center (18S rRNA; G1408 and A1491) (42). This reduced affinity forms part of the basis for their selectivity for the prokaryotic ribosome. Nonetheless, a subset of aminoglycosides has been shown to weakly bind eukaryotic ribosomes in a manner sufficient to disrupt the normal proofreading function of the ribosome leading to an increase in the insertion of a near-cognate aminoacyl-tRNA in the ribosomal A-site (43). For our discussion here, it is important to note that the reduction in translation fidelity occurs at both sense and nonsense codons.

The general reduction in translation fidelity has proved to be particularly useful in terms of promoting nonsense suppression at PTCs. Of note, before the biochemical mechanism of aminoglycoside antibacterial activity was known, aminoglycosides were shown to possess the capacity to suppress PTCs and lead to the production of full-length protein. This property was first demonstrated for the aminoglycoside streptomycin when in 1964 Gorini and Kataja (44) showed the phenotypic correction of defective genotypes induced by PTCs in *Escherichia coli*. Extending upon these initial observations, the nonsense suppressive activity of aminoglycosides, including gentamicin, amikacin, paromomycin, geneticin (G418), lividomycin and tobramycin, has now been demonstrated in numerous cell lines and cell free extracts, including those derived from patients with various genetic disorders (6,45–50). The success of aminoglycosides in cell culture and mouse models of human disease quickly prompted their testing in patients with disease caused by nonsense mutations. Remarkably, the administration of gentamicin has been shown to promote partial restoration of full-length functional protein in clinical trials for a variety of diseases, including cystic fibrosis (CF) (51), Duchenne muscular dystrophy (DMD) (52), hemophilia A and B (53), and Hailey-Hailey disease (54). For instance, the intranasal administration of gentamicin to the nasal mucosa for 14 days in nonsense mutation CF patients has been shown to result in local CF transmembrane conductance regulator (CFTR) protein production and an improvement in chloride channel activity (51). Additionally, intravenous gentamicin administration in patients with nonsense mutation DMD resulted in an increase in full-length dystrophin in muscle biopsies (52). While these studies are promising it should be noted that not all patients enrolled in these studies responded positively to treatment.

Although aminoglycosides clearly exhibit nonsense suppressive properties, there are several obstacles that must be overcome before they can be used for long-term suppressive therapy in patients. For one, the efficiency at which aminoglycosides can suppress PTCs is significantly affected by the identity of the termination codon. For example, the efficiency of gentamicin for suppressing various PTCs has been shown to vary greatly (5,50). Additionally the context of the PTC, both 5′ and 3′ nucleotides, also influences the efficiency of suppression, with the presence of a cytosine in the +1 position (nucleotide immediately 3′ of the termination codon) promoting the highest levels of basal and drug-induced suppression (5,55). This suggests that only a subset of PTC-carrying patients would be likely to benefit from aminoglycoside treatment regimens.

Perhaps more troublesome is the fact that aminoglycosides do exhibit significant toxicity (56,57). One way that aminoglycosides enter cells is through the receptor, megalin (58). Megalin is a multi-ligand endocytic receptor that is highly expressed in the proximal tubules of the kidney and the cochlear hair cells of the inner ear, resulting in the accumulation of aminoglycosides in these two locations. In line with this, two of the more common complications of aminoglycoside treatment are nephrotoxicity and ototoxicity (59–61). While the cellular toxicity is likely due to many contributing factors, three distinct mechanisms have been proposed. First, following endocytosis, aminoglycosides become positively charged, allowing them to interact with phospholipids and interfere with phospholipase signaling occurring on the lysosomal membrane (62). Secondly, due to their charged nature, aminoglycosides have been shown to lead to the generation of reactive oxygen species (ROS) (63). Finally, in individuals with specific polymorphisms in their mitochondrial 12S rRNA, aminoglycosides can interact with the mitochondrial ribosome resulting in mitochondrial dysfunction (64). In conclusion, the toxicity associated with aminoglycosides in conjunction with their specificity for specific nonsense codons (and flanking nucleotides) prevents their long-term use in all patients with nonsense mutation-mediated disease. Additionally, as aminoglycosides work by generally reducing the fidelity of proofreading, there is significant potential to disrupt normal decoding at both sense and nonsense codons. These issues highlight the importance of identifying additional small molecules that are capable of modulating the efficiency of translation termination.

### PTC124

In an effort to identify novel small molecules capable of selectively suppressing PTCs, Welch *et al* (65) screened ~800,000 low molecular weight compounds for their ability to specifically suppress a PTC within an integrated luciferase gene in HEK293 cells, while not affecting termination at the normal stop codon. Through these screens PTC124 {3-[5-(2-fluorophenyl)-[1,2,4]oxadiazol-3-yl]-benzoic acid; C_15_H_9_FN_2_O_3_} was identified (Fig. 2). Of note, PTC124 lacks any structural similarity to aminoglycosides or other clinically developed drugs.

The initial description of PTC124 demonstrated dose-dependent read-through of all three stop codons. Remarkably, PTC124 was more efficient than aminoglycosides at promoting read-through. Specifically, low concentrations (0.01–10 μM) of PTC124 promoted significant PTC suppression in tissue culture, whereas 100 μM gentamicin failed to exhibit any read-through. Furthermore, while aminoglycosides are known to globally reduce translation fidelity and will thus affect translation termination at normal termination codons, PTC124 demonstrated specificity for the PTC within the luciferase open reading frame (ORF). Additionally, global protein and mRNA profiles appear unaffected by PTC124 (65).

In addition to demonstrating the nonsense suppressive activity of PTC124 in tissue culture, Welch *et al* (65) also demonstrated the utility of PTC124 in the *mdx* mouse model of nonsense mutation DMD. Using a treatment regimen that targeted a plasma concentration of 5–10 μg/ml, treatment with PTC124 was able to improve multiple phenotypes, including a functional strength deficit, protection against contraction-induced injury, and a reduction in serum creatine kinase levels. Accordingly, western blot analyses demonstrated a 20–25% increase in dystrophin levels in animals treated with PTC124. To date, PTC124 has been tested preclinically in diverse models of nonsense-mediated disease, including CF (66), Miyoshi myopathy (67), Hurler syndrome (6), Carnitine palmitoyltransferase 1A deficiency (68), Usher syndrome (69) and Batten disease (70).

PTC124 has gone through phase I clinical trials where it has been deemed safe for therapeutic uses (71). Consistent with the preclinical animal data, phase II clinical trials for CF and DMD both reported positive findings. For instance, CF patients treated with PTC124 for three months exhibited increased chloride channel activity, as well as an improvement in pulmonary function (72,73). Additionally, in a separate nonsense mutation DMD phase II study, the oral administration of PTC124 increased dystrophin protein expression in 61% of the patients (74). It is currently in phase III clinical trials for both CF and DMD (75; http://clinicaltrials.gov/ct2/results?term=ataluren).

Currently, the mechanism by which PTC124 promotes PTC selective nonsense suppression is unknown. For normal translation termination to be unaffected by PTC124 it would suggest that termination at a PTC is mechanistically different from termination at a normal stop codon. Of note, consistent with this notion, ribosomal toe-prints are much more pronounced at PTCs than they are at normal stop codons (76). This suggests that translation termination at a PTC is kinetically less efficient and that ribosomes pause for a greater amount of time at PTCs than for normal termination codons. Exactly how and whether this matters with regard to the mechanism of PTC124-mediated nonsense suppression is unclear. Undoubtedly, it will be interesting to determine the mechanism of its nonsense suppressive activity.

Although the results of PTC124 have been nothing short of remarkable, it has not gone without some controversy. Initial concerns were primarily derived from the setup of the high throughput screen that identified PTC124. As mentioned, the screen utilized a luciferase construct containing a PTC that prevented the synthesis of full-length firefly luciferase (FLuc) protein (65). The logic behind this assay is that suppression of the PTC would generate full-length luciferase which can then be detected by an increase in luminescence. However, shortly following the initial description of PTC124, it was demonstrated that PTC124 interacts with ATP generating the stable acyl-AMP mixed-anhydride adduct PTC124-AMP (77,78). When bound to FLuc it results in its stabilization and an increase in steady-state luciferase activity, which in Welch’s screen (65) would score as a molecule with nonsense suppressive activity. Interestingly, this interaction is specific for FLuc as no stabilization was seen for *Renilla* reniformis luciferase (RLuc). In line with the hypothesis that PTC124 simply stabilized FLuc thereby promoting an increase in luminescence, PTC124 failed to promote nonsense suppression of an RLuc reporter (77). It is important to note that rebuttals against the conclusion of this study have been published (79). More recently, McElroy *et al* (80) also failed to detect the nonsense suppressive activity for PTC124 using a variety of reporter systems. Regardless of the role of off-target effects of PTC124 on FLuc activity during its initial identification, the fact is PTC124 has demonstrated positive results in multiple preclinical models of disease, as well as in clinical trials involving patients with nonsense mutation disease. The ability to promote functional full-length protein expression in patients with nonsense mutation disease is exactly what PTC124 was developed to do. Thus, PTC124 represents a potential pharmacological PTC suppression therapy that could reduce disease severity for many patients and certainly warrants further investigation in to its mechanism of action as well as the breadth of patients that may benefit from its use as a therapy.

### Pseudouridine (ψ)-mediated suppression

**Figure 3:**
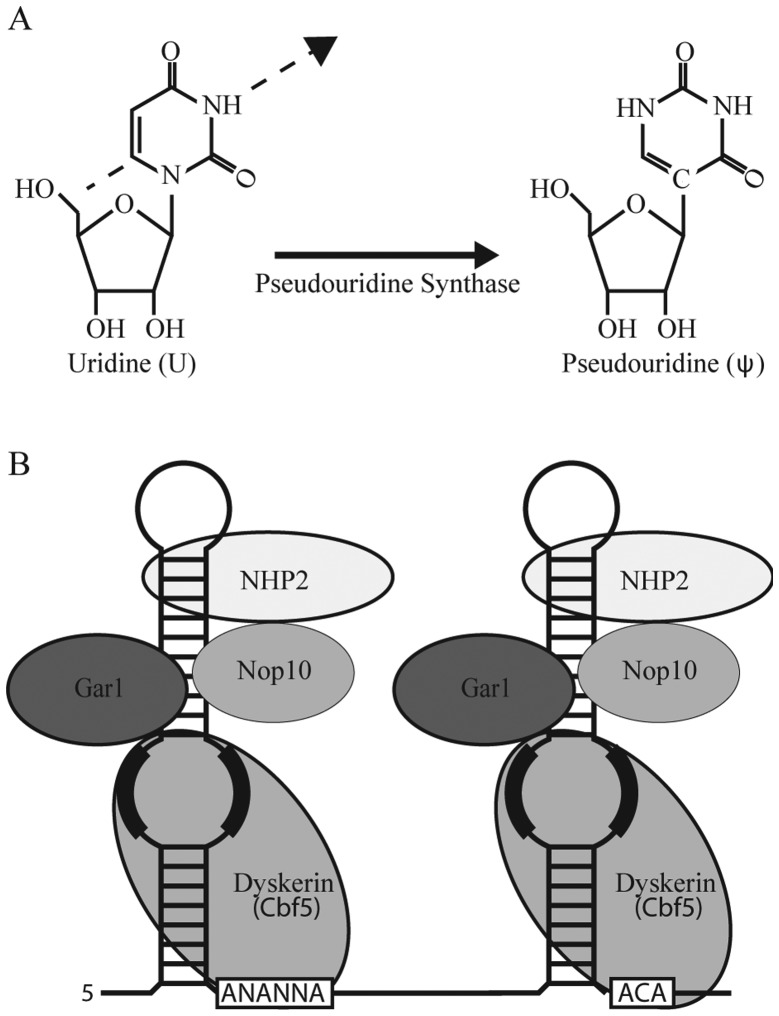
Box H/ACA ribonucleoproteins (RNPs) catalyze pseudouridylation. (A) Pseudouridine is the 5-ribosyl isomer of uridine. Dashed arrow represents the rotational axis. (B) Box H/ACA RNPs consist of four core proteins, namely Dyskerin, Gar1, Nhp2 and Nop10. Dyskerin is the pseudouridine synthease. Cbf5 is the Dyskerin homologue in yeast.

In addition to the development of small molecule-based approaches to promoting nonsense suppression, recently, we have been successful in developing a novel strategy that specifically targets the PTC for recoding through nucleotide modification (81). Post-transcriptional nucleotide modifications are naturally abundant in various cellular RNAs. Pseudouridine is the C-5 glycoside isomer of uridine and is particularly abundant in rRNA and the spliceosomal U small nuclear RNA (snRNA) (Fig. 3A). Generally speaking, ψ optimizes the structure and function of rRNAs and U snRNAs in translation and pre-mRNA splicing, respectively (82,83). It is well established that ψ has distinct biochemical properties from uridine. In each case these biochemical properties depend on the structural context and can extend beyond the site of modification. For instance, multiple studies have demonstrated that RNA fragments containing ψ are significantly more stable than if the same RNA contained uridine (84).

The fact that ψ is biochemical distinct from uridine, coupled with the fact that a uridine is present at the first position of all termination codons prompted us to test whether the incorporation of a ψ into a termination codon could affect translation termination *in vitro* (81). Remarkably, replacing the uridine residue with a ψ drastically reduced the efficiency of translation termination in both rabbit reticulocyte lysate and *Escherichia coli* lysate (81,85). With the success of our *in vitro* translation system, we set forth towards developing means to target PTC containing transcripts in cells.

One mechanism by which pseudouridine can be generated is through the action of Box H/ACA ribonucleoprotein (RNP) complexes (86) (Fig. 3B). Box H/ACA RNPs consist of 4 core protein components, one of which is a pseudouridine synthase, and a non-coding RNA referred to as a Box H/ACA RNA (Fig. 3B). While the protein components of the RNP are responsible for stabilization of the RNP as well as enzymatic activity, the non-coding RNA is responsible for substrate recognition through complementary base pairing interactions. Thus, in theory, through the construction and expression of artificial Box H/ACA RNAs any uridine residue should be amenable to targeted modification (87).

Taking advantage of the fact that one can ‘design’ novel Box H/ACA RNAs to target any RNA, we developed a system to specifically detect pseudouridine-mediated nonsense suppression *in vivo* (in *S. cerevisiae*), namely the *CUP1-PTC* reporter system (81). The *CUP1-PTC* reporter system utilizes the CUP1 gene which provides cells with resistance to copper; hence the introduction of a PTC into the CUP1 gene renders cells sensitive to copper. Remarkably, while cells expressing a control box H/ACA RNA were unable to grow on media containing copper, expression of a box H/ACA RNA targeting the PTC of the *CUP1-PTC* transcript restored cellular growth (albeit partially). Additionally, targeted pseudouridylation of PTCs located within the yeast TRM4 gene similarly resulted in nonsense suppression as determined by western blot analysis of full-length protein. Interestingly, immunoprecipitation coupled with mass spectrometry determined that the pseudouridylated termination codons of TRM4 resulted in the incorporation of specific amino acids. Specifically, ψAG and ψAA both resulted in the corporation of serine and threonine, while ψGA directed the incorporation of tyrosine and phenylalanine (81). As the biochemical properties of ψ are largely affected by its context, whether the local nucleotide context of the pseudouridylated termination codon influences which amino acid is incorporated is an interesting idea that deserves more attention.

Recently. high resolution X-ray crystallographic data has shed some light on the mechanism by which pseudouridine is able to promote nonsense suppression (85). For example, during the normal translation decoding process, the tRNA-mRNA base pair interaction is monitored by A1493 of the ribosomal decoding center. This nucleotide normally adopts the *anti* conformation; however, the decoding of ψAG by tRNA^Ser^ results in A1493 adopting the *syn* conformation. Further non-canonical interactions between the ψAG and the anticodon of tRNA^Ser^ were observed, including normally forbidden purine-purine base pairs at the second and third positions. Interestingly, these interactions were mediated by an unusual Watson-Crick/Hoogsteen geometry. Thus, the ribosome appears to be capable of accommodating non-canonical base pairs. Ultimately, determining the effect of ψ on the rate constants of various steps in decoding, and on the efficiency of termination will help to clarify the mechanism of pseudouridine-mediated nonsense suppression.

While site-specific targeted pseudouridylation represents a novel means of promoting nonsense suppression and offers both unprecedented specificity and versatility, it too faces great obstacles in terms of being clinically relevant. The greatest challenge is how one would introduce the guide RNAs into cells in a manner that would allow them to still function, as well as protect them from recognition by the cellular innate immune system RNA sensors (88). Additionally, it is unknown whether all mRNA molecules will transit through cellular locations that make them susceptible to efficient box H/ACA RNA-mediated pseudouridylation.

## 4. Conclusion

PTCs can arise from a variety of mutations in either germ or somatic cells. Overall, an estimated one third of all genetic disorders are caused by PTCs (89). As the genes and specific mutations that cause various diseases continue to be identified, there is no doubt that more disease phenotypes will be attributed to the presence of PTCs. Thus the need to develop drugs that are capable of promoting nonsense suppression will only grow.

To date, therapeutic PTC suppression has been demonstrated in both preclinical animal models and in clinical trials for diseases such as CF and DMD. The suppression of PTCs with small molecules represents an approach to treat nonsense mutation disease that is growing in favor. In addition to small molecule approaches strategies that aim to promote translational read-through via targeted nucleotide modification of the PTC, such as the recent discovery of ψ-mediated nonsense suppression, are also being explored. It is unclear whether a single therapeutic strategy will suffice to promote nonsense suppression at any PTC or whether therapeutic PTC suppression will require a case by case analysis. Nonetheless, with the continuing accumulation of mechanistic insight into translation termination the field is certainly poised to develop novel therapeutics.
